# A Right Ventricular Mass With Intracavitary Obliteration: Tumor or Thrombus?

**DOI:** 10.7759/cureus.50809

**Published:** 2023-12-19

**Authors:** Ozer Kandemir, Emre Boysan, Renda Circi, Tugce Turker, Ferit Cicekcioglu

**Affiliations:** 1 Cardiovascular Surgery, Etlik Şehir Hastanesi, Ankara, TUR

**Keywords:** differential diagnosis, spindle cell neoplasms, cardiac mass tumor, rv thrombus, right ventricular cardiac mass

## Abstract

A 47-year-old woman was admitted to the hospital because of dyspnea for the past three months. She was previously diagnosed with pulmonary embolism. She had been operated on for a colon tumor five years ago and no residual cancer was detected on oncological follow-up. Her transthoracic echocardiographic and transesophageal echocardiographic evaluation showed a hypertrophic right ventricle occupied by a 2.7 x 4.8 cm immobile mass obliterated to the right ventricle cavity. All the non-invasive tests were consistent with thrombus prediagnosis. She underwent surgery. Mass was resected from the right ventricle as much as possible. Histopathology of surgical material revealed metastatic spindle cell adenocarcinoma.

We aim to increase the awareness of the differential diagnosis of thrombus or tumor, thereby leading to appropriate management.

## Introduction

Primary and secondary cardiac tumors, thrombus, cysts, and vegetations are the most common reasons for cardiac masses [1]. Right-sided cardiac masses are rare and do not have a uniform clinical presentation [2].

An autopsy series found right atrial (RA) and right ventricular (RV) thrombus in 3.1% and 0.5% of cases, respectively [3,4]. The majority of right-sided cardiac masses that occupy the right ventricle may give symptoms of right heart failure, potentially leading to hepatomegaly and systemic edema. However, patients may be asymptomatic before complications such as pulmonary embolism and paradoxical stroke develop [3,5]. The most common reasons for RV thrombus are venous thromboembolism, catheters, and pacemaker leads, or due to the stasis of blood in atrial fibrillation (AF) and cardiomyopathies [3].

A primary cardiac tumor is extremely low in occurrence, accounting for 0.001-0.03% of all cardiac malignancies. Metastatic tumors are 30 times more common than primary tumors of the heart [6,7].

A mass seen in the right ventricle must be diagnosed before treatment because the treatment varies according to the diagnosis.

In this case report, we present a patient who had a cardiac mass that covered almost the entire right ventricle and had been operated with prediagnosis as the cardiac thrombus.

## Case presentation

A 47-year-old woman was admitted to the hospital because of dyspnea for the past three months. She was diagnosed with pulmonary embolism three times previously, which was confirmed by computed tomography (CT). No mass formation on the right side of the heart was observed. She received warfarin therapy for three months. On her physical examination, she had mild respiratory distress and normal cardio-respiratory findings. Her vital signs were blood pressure of 100/60 mmHg, pulse rate of 95 beats per minute, and partial oxygen concentration of 95% on 3 L/minute oxygen via nasal cannula.

She had been operated on for a colon tumor five years ago, which was pathologically diagnosed as spindle cell adenocarcinoma. She had received chemotherapy via port access and no residual cancer was detected on oncological follow-up.

**Figure 1:**
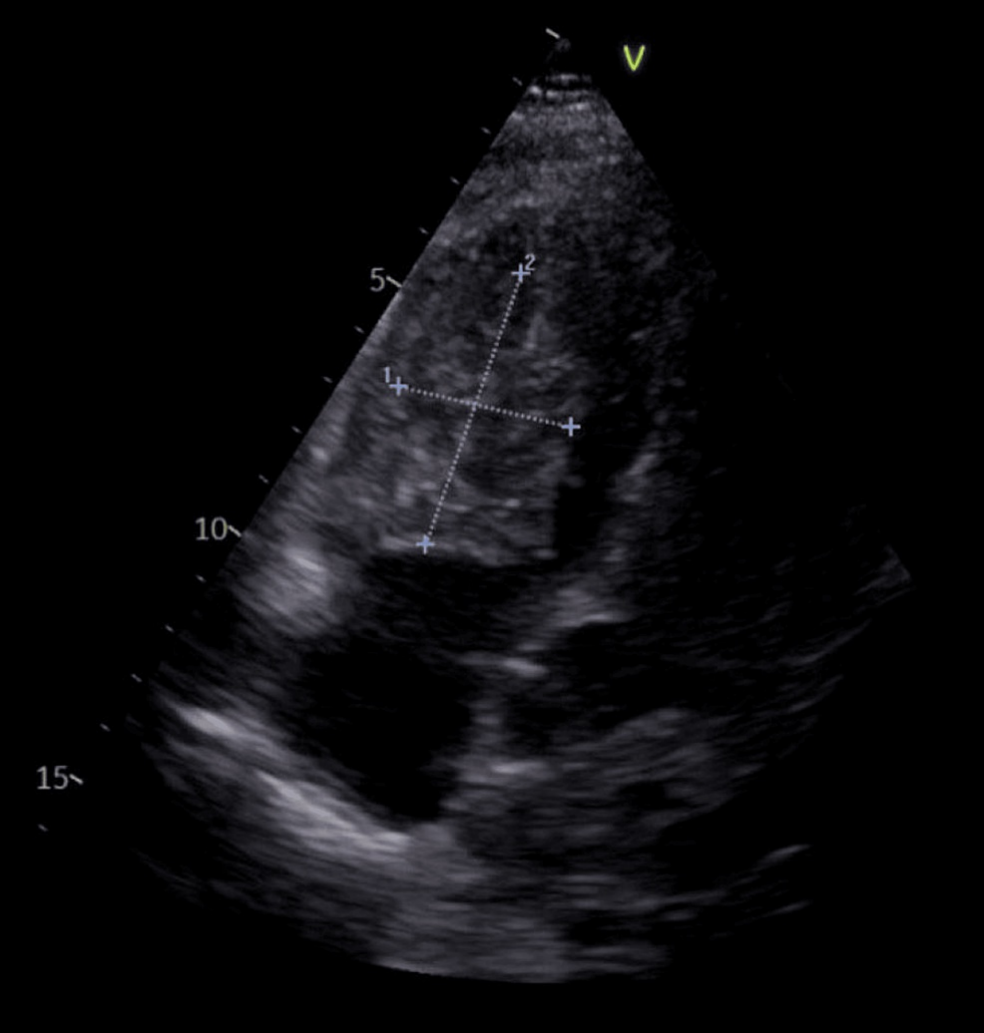
Transthoracic echocardiographic image of the mass.

She had no history of cardiovascular disease. Her echocardiographic evaluation showed that the hypertrophic right ventricle was occupied by a huge (2.7 x 4.8 cm), immobile mass attached to the interventricular septum (Figure 1). The distal end of the port access was detected in the right atrium with no evidence of thrombus. There was no stenosis or regurgitation on the tricuspid valve. RV ejection fraction and tricuspid annular plane systolic excursion (TAPSE) were slightly depressed (40% and 12).

A giant thrombus located in the right ventricle triggered by port access was considered a preliminary diagnosis. Although no relapse was considered as a result of the oncology consultation, it was decided to perform cardiac CT and magnetic resonance imaging (MRI) for the differential diagnosis of the tumor.

A cardiac CT was performed. An appearance compatible with a thrombus covering almost the entire right ventricle was detected.

**Figure 2:**
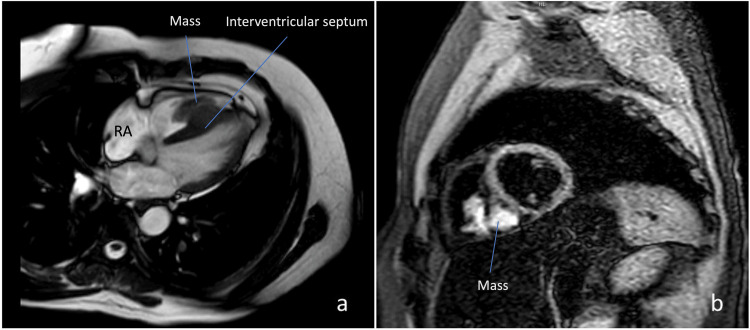
Magnetic resonance imaging of the mass attached to the interventricular septum (PSIR and sBTFE sequences). RA: right atrial; PSIR: phase-sensitive inversion recovery; sBTFE: saturation recovery with balanced turbo field echo pulse.

Also, a cardiac MRI was performed for differential diagnoses. No contrast detected any sequence of magnetic resonance in favor of the tumor. Similar to echocardiography and cardiac CT, a right ventricular mass with intracavitary obliteration was evaluated as a thrombus (Figure 2).

We also performed a PET-CT. A low standardized uptake value (SUV) was detected, which favors to thrombus.

Although RV functions were depressed, it was decided to perform surgery due to frequent pulmonary embolism attacks and the fact that the RV mass was compatible with the thrombus and there was no response to anticoagulant treatment.

**Figure 3:**
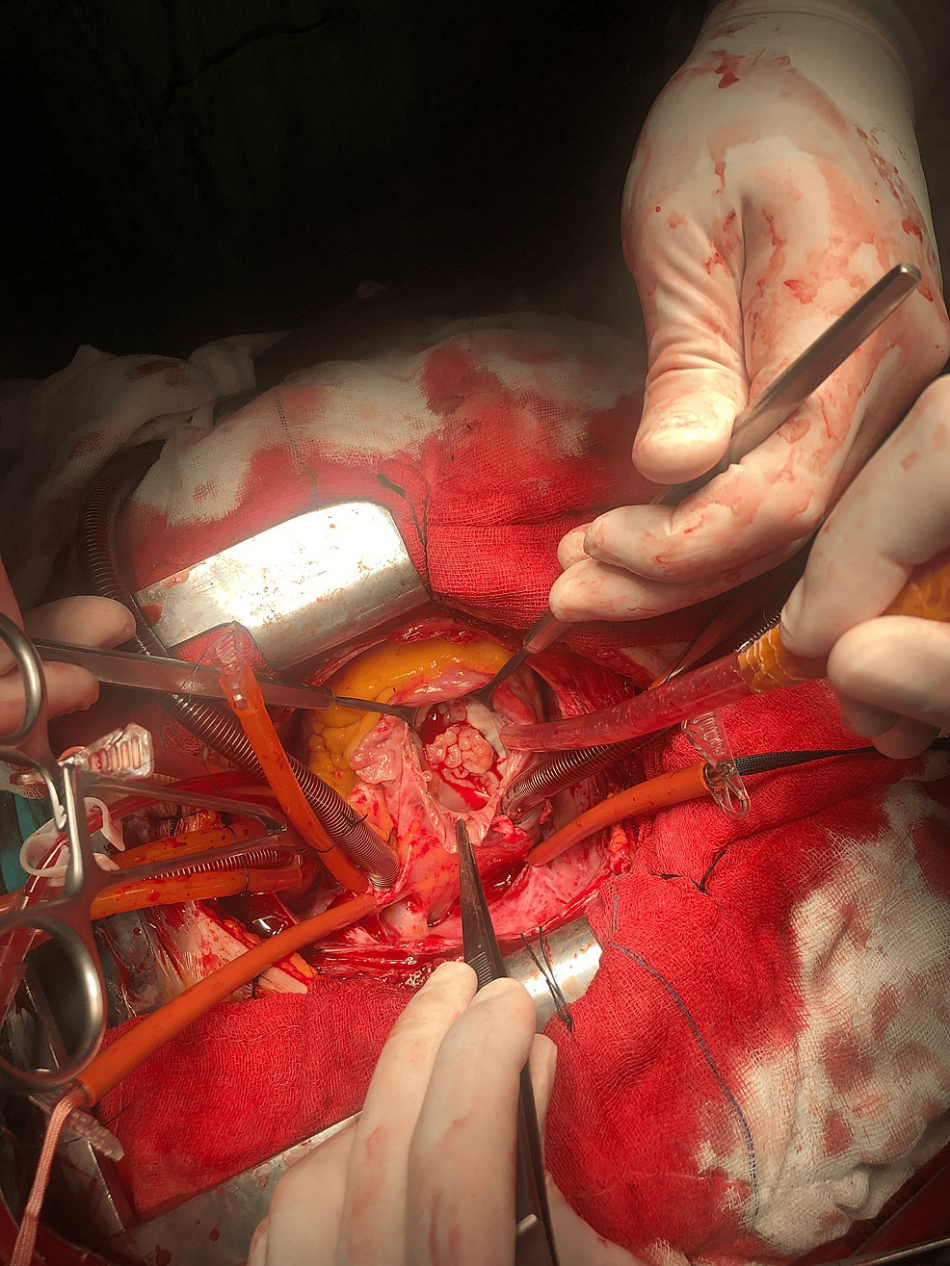
Intraoperative view of the mass through the right atrium.

She underwent surgery. After median sternotomy, aorto-bicaval cannulation was performed. X-clamp was placed and antegrade blood cardioplegia was given for diastolic arrest. RV was severely hypertrophic. The right atrium was opened. Heterogeneous fragile right ventricle mass prolapsed from the tricuspid valve to the right atrium was seen (Figure 3).

**Figure 4:**
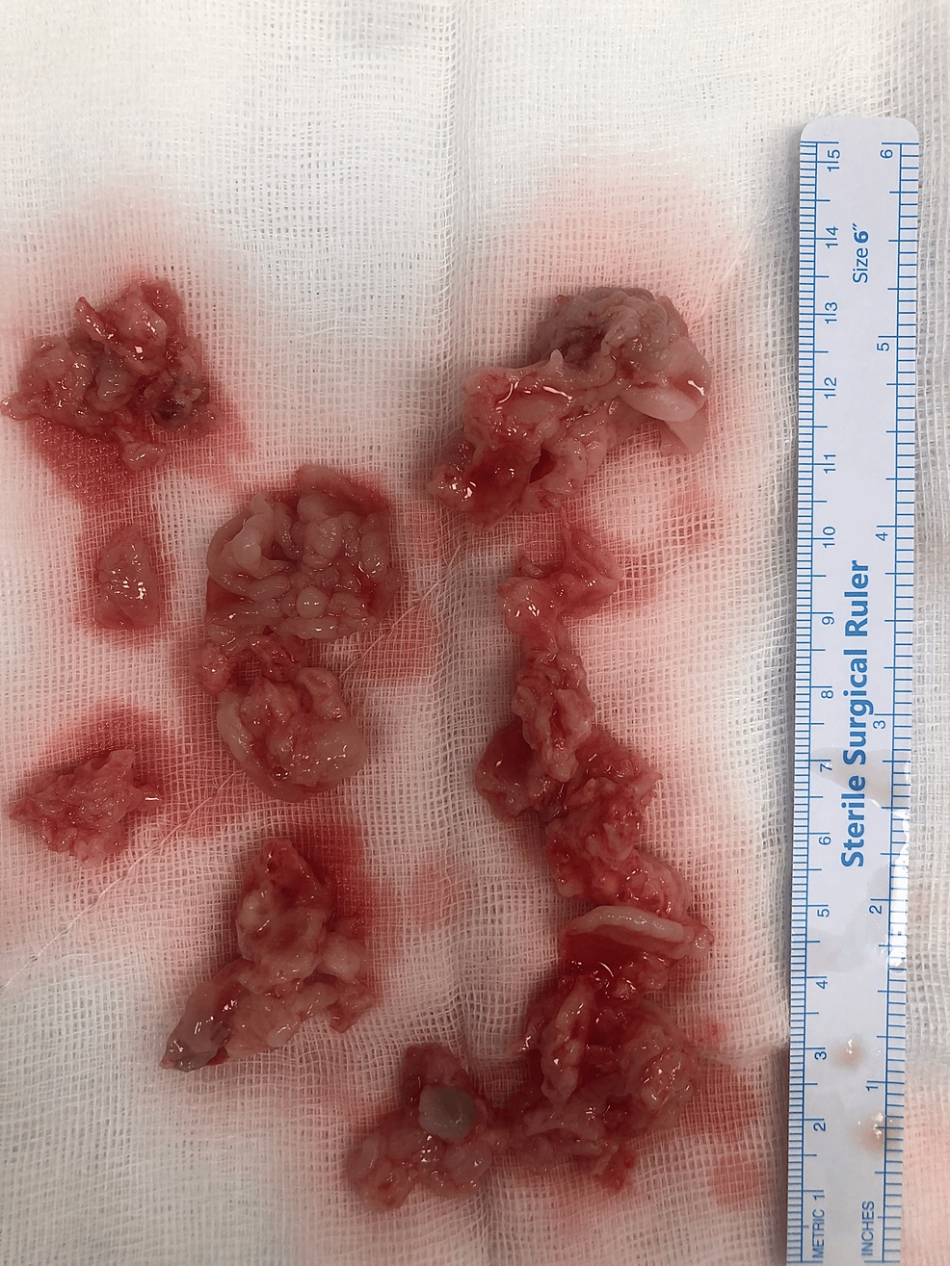
The appearance of the mass after resection.

The mass was obliterated in the right ventricle, and also filled the posterior regions of all leaflets, preventing tricuspid valve movements. The masses that could be reached in the RV were cleaned. It was heterogeneous and fragile (Figure 4). The septal and anterior leaflets of the tricuspid valve were separated from the annulus and the mass structures, which attached chordae, and the posterior of the leaflets were cleaned. Then the leaflets were stitched back into the annulus. The cardiopulmonary bypass was finished uneventfully with inotropic support. However, the patient expired due to right ventricular failure on the sixth day postoperatively.

Histopathology of surgical material revealed metastatic spindle cell carcinoma.

## Discussion

Cardiac masses can be seen due to different reasons such as thrombus, primary and secondary tumors, cysts, and vegetation [1]. Right-sided cardiac masses are infrequent and do not have a uniform clinical presentation [2].

Ögren et al. found RA and RV thrombus in 3.1% and 0.5% of cases in an autopsy series, respectively [4]. Predisposing factors of right-sided thrombus included venous thromboembolism, medical devices such as catheters and pacemaker leads, or due to the stasis of blood in AF and cardiomyopathy [3]. Malignancy is the other important cause of right-sided thrombus via tumor secretion of pro-coagulant tissue factors and increased platelet activity [8]. A right-sided thrombus may have lethal complications, such as pulmonary embolism and paradoxical stroke. Five-year mortality in the right-sided cardiac thrombus population was high (32/97, 33.0%) despite a mean left ventricle ejection fraction of 47.9% [3].

Primary cardiac tumor frequencies were reported in 0.001-0.03% of all cardiac malignancies [6,7]. Metastatic tumors are seen 30 times more commonly than primary cardiac tumors [7]. Lung cancer is the most common primary tumor that has the highest incidence of cardiac metastasis, followed by hematological malignancies and breast cancer [6]. Other tumors with high rates of cardiac metastasis include ovarian, renal, pancreatic, and gastric carcinomas [9,10]. Malignant melanoma has the highest tendency to metastasize to the heart but the overall incidence is low [9]. Tumors can reach the heart via four pathways: hematogenous spread, lymphatic spread, transvenous, and direct extension [10]. Cardiac metastases may lead to pulmonary embolism in 3%-26% of cases [11]. Autopsy reports show that up to 26% of patients who die of cancer have tumor cells in their pulmonary vasculature [12].

Differential diagnosis is very important, whether it is a thrombus or tumor. For this distinction, non-invasive evaluation of cardiac masses should be performed, such as transthoracic and transesophageal echocardiography, cardiac tomography, and cardiac MRI. Echocardiography informs about the thrombus size, location, and mobility. Thrombus is avascular and never has a stalk [6]. Immobile and giant thrombus located close to the wall can mimic a tumor. A wall motion abnormality, global hypokinesis, and autoimmune condition may also favor thrombosis [13]. Cardiac CT and cardiac MRI also inform about the anatomic localization, calcification, and vascularity of the mass. The contrast of the mass in the CT or MRI is interpreted in favor of the tumor. For uncertain and diagnostically challenging cases, PET-CT and guided biopsy of the mass should be done.

In our case, all the non-invasive tests we performed concluded that the mass in the RV was thrombi. MRI and PET-CT showed no involvement or contrast enhancement in favor of any tumor.

Surgery was decided due to frequent pulmonary embolism attacks despite anticoagulant treatment, increasing complaints of shortness of breath, the young age of the patient, and the absence of any examination suggestive of a tumor. Surgical treatment aimed to remove the mass in the RV to relieve the symptoms of the patient, prevent pulmonary embolism attacks, and perform a pathological examination for a definitive diagnosis.

The average life expectancy in patients with cardiac metastases is less than six months [7,14]. There is no guideline on how to treat such patients. Despite the poor prognosis, there are also publications stating that tumor resection with open heart surgery should be performed in such patients. It is stated that tumor resection prolongs life expectancy, improves the clinical picture, and prevents tumor and/or thrombus embolism [7,15].

## Conclusions

Although RV metastatic tumors are rare, they should be kept in mind. Even if non-invasive tests do not support it, invasive biopsy should be kept in mind, especially in patients with a history of cancer.

Through this article, we aim to increase the awareness of the differential diagnosis of thrombus or tumor, thereby leading to appropriate management.
